# A report of presacral epidermoid cyst in perimenopausal women: An extremely rare site and an unusual cause of chronic constipation

**DOI:** 10.1016/j.ijscr.2023.107880

**Published:** 2023-01-09

**Authors:** Salem M. Tos, Afnan W.M. Jobran, Anas Alasafrah, Izzeddin Bakri, Fahmi Jubran

**Affiliations:** a Faculty of Medicine, Al Quds University, Jerusalem, Palestine; b General Surgeon, Al Ahli Hospital, Hebron, Palestine; c Pathology department, Al Ahli hospital, Hebron, Palestine; d Al Ahli Hospital, Hebron, Palestine

**Keywords:** Pelvic mass, Presacral, Epidermoid cyst, Case report

## Abstract

**Background:**

Epidermoid cyst is rare congenital lesion of ectodermal origin that arises from the remnants of the embryonic tissues. Although epidermal cysts are frequently observed throughout the body, they are rarely found in the presacral regions. It more commonly occurs in women of reproductive age as an asymptomatic, incidental finding during routine physical examination or imaging studies, or during obstetric and gynecologic events.

**Case presentation:**

A 48-year-old female patient presented with intermittent constipation, lower abdominal and pelvic pain that had developed progressively during the previous six months, which was temporarily relieved with regular laxatives. Magnetic resonance imaging showed a presacral cystic tumor with a high signal intensity on T1-weighted images and, low signal on T2-weighted images with no significant enhancement post-contrast administration. The mass pushed the rectum laterally by external compression. This tumor was diagnosed as a developmental cyst, and total mass resection with negative margins was performed. After that, tumor was histopathologically diagnosed as an epidermoid cyst.

**Discussion and conclusion:**

Epidermoid cyst is a common entity but is rare at presacral space. Also, an epidermoid cyst is an uncommon entity among cystic pathologies found in the presacral region, which includes benign and malignant pathologies. Due to the potential of subsequent infection or cancer, a meticulous clinical examination with correlated radiographic imaging, followed by total mass excision and histopathological evaluation are crucial.

## Highlights

• Epidermal inclusion cysts, simply called epidermoid cysts, are very rare benign lesion that develops from an ectodermal tissue remnant.
• Epidermoid cysts can be seen all over the body, but are very rare in the presacral region with an incidence of 0.0025 %.
• Careful history taking, meticulous physical examination, and investigations including imaging and biopsy are necessary.
• Prompt treatment should be addressed in a proper way to stabilize the patient and avoid mortality.

## 1 Introduction

Epidermal inclusion cysts, simple called epidermoid cysts, are very rare benign lesion that develops from an ectodermal tissue remnant and thus have a squamous epithelial lining, which is misplaced during embryogenesis due to faulty development of adjacent structures [1], [2]. Epidermoid cysts can be seen all over the body, but are very rare in the presacral region with an incidence of 0.0025 % [2]. They have generally been reported in asymptomatic women as incidental findings [3] during routine physical examination or imaging studies, or during obstetric and gynecologic events [4], [5], [6], [7]. Some reported cases presented with symptoms related to local compression on the rectum causing constipation, rectal fullness, painful defecation, and lower abdominal pain, or on the lower urinary tract, which causes dysuria and urinary frequency [1]. Rectal masses or recurrent perianal abscess and sinus are other reported clinical pictures [8]. Also, an epidermoid cyst is an uncommon entity among cystic pathologies found in the presacral region, which includes benign tumors (e.g epidermoid, dermoid, and enteric cysts) and malignant pathologies (e.g teratoma, teratocarcinoma, and yolk sac tumor) [1]. It is recommended that these lesions be totally excised because of the risk of secondary infection, malignancy or pregnancy complications.

Herein, we present a very rare case of a large epidermoid cyst in the presacral region presented as chronic constipation in perimenopausal women. It was diagnosed using clinical findings, Correlated radiographic imaging, and histopathology with a successful removal via total mass excision.

This work has been reported in line with the SCARE criteria, which are used by authors, journal editors, and reviewers to increase the robustness and transparency in reporting surgical cases [16].

## 2 Case presentation

A 48-year-old female patient with no medical history presented with lower abdnominal and pelvic pain that had developed progressively during the previous six months. She had also experienced intermittent constipation for the same period, which was temporarily relieved with regular laxatives. The patient's surgical history was anal fissure which were treated via lateral internal sphincterotomy five years ago. The patient denied other gastrointestinal symptoms. The reproductive and urinary tract were also unremarkable. She also denied lower limbs numbness, tingling, weakness, paresthesias, or recent trauma. Upon examination, she was vitally stable and no obvious or significant signs were present.

### 2.1 Workup

The laboratory findings including hemoglobin level, leukocyte count, platelet count, blood urea nitrogen level, and creatinine level all were within the normal ranges. The serum levels of tumor markers (CA 15–3, CEA, CA 125) were also within normal ranges.

**Fig. 1:**
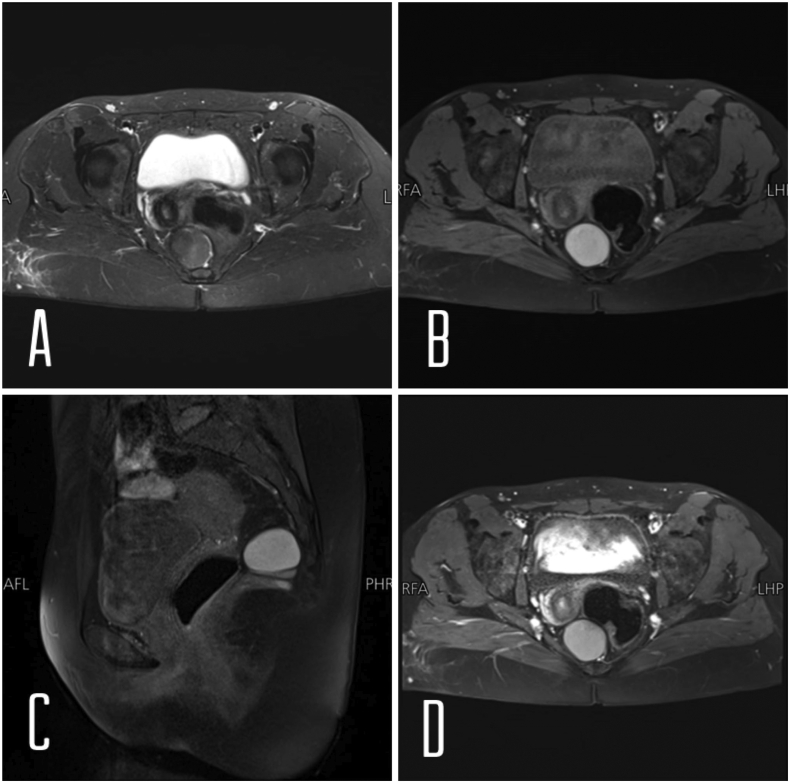
Magnetic resonance imaging (MRI) showed entirely capsulated cystic mass which was located in the right dorsal side of the rectum . The main bulk of the lesion appears hypo-intense on T2-weighted imaging (A), but homogeneously hyper-intense on T1-weighted imaging (B and C) with no significant enhancement post contrast administration (D).

Contrast-enhanced abdominal CT showed a 4 × 5-cm hypodense cystic lesion pressing externally on the rectum in the presacral region. Magnetic resonance imaging (MRI) showed a 4 × 4 × 5-cm entirely capsulated multi-loculated cystic lesion seen in the presacral region posterior to the rectum with no internal septations. The main bulk of the lesion appears hypo-intense on T2-weighted imaging, but heterogeneously hyper-intense on T1-weighted imaging with no significant enhancement post-contrast administration (Fig. 1). Tiny T1 and T2 hypo-intense focus seen at the dome of the lesion could represent wall calcification. The main bulk of the lesion shows true diffusion restriction in Diffusion-Weighted imaging (DWI) and Apparent Diffusion Coefficient (ADC). Tiny adjacent peripheral cysts are also seen. The mass pushed the rectum laterally by external compression.

Multiple solid lesions are also seen related to myometrium mostly representing uterine fibroids, the largest measuring 3 cm showed subendometrial extension located at the anterior uterine wall. Smaller intramural and subserosal fibroids are also seen. Both ovaries are not well identified and could be atrophied in such patients with early menopause. Small subcentimetric pelvic lymph nodes were also present.

Colonoscopy provided a clear image of the mass compressing the external wall of the rectum. Biopsies of rectal mucosa collected during the colonoscopy yielded no specific findings.

After a discussion with the patient and the family about the differential diagnosis and management options, the decision was made to proceed with total mass excision.

### 2.2 Intraoperative

The patient was taken to the operating room. While the patient was under general anesthesia and placed in the supine position with the head of the bed elevated at 15°, a low transverse (Pfannenstiel) incision of 8 cm in length was made, followed by the opening of the patient's anterior abdominal wall in layers. The mass was found dorsal to the rectum and was circumferentially dissected away from the muscles within the presacral space and with the preservation of the adjacent structures. After the excision, a rectal examination was performed confirming that there was no entry into the rectal lumen. The anterior abdominal muscles were then reapproximated and the skin closed. The patient tolerated the procedure well without complication.

### 2.3 Gross examination

Received a single yellowish-white well-defined nodule, measuring about 4 × 5 cm that was firm to hard in consistency and it was filled with mucoid, yellowish-colored fluid.

### 2.4 Histopathology

**Fig. 2:**
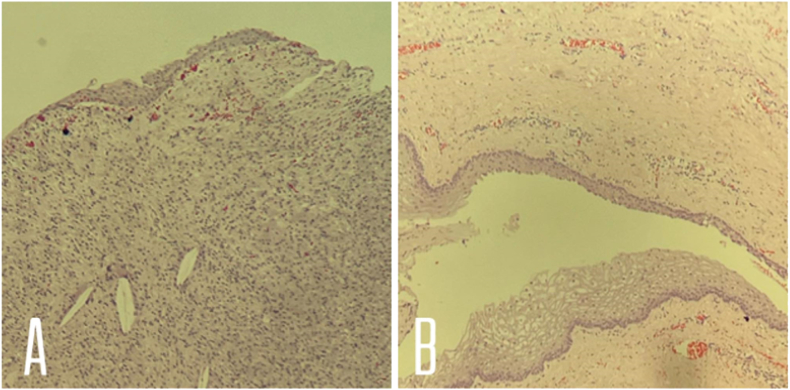
Sections of an epidermoid cyst showed: (A) a cystic structure occupying the entire dermis. The lesion is unilocular .The lining of the cyst composed of an epithelium which is flattened and contains a granular layer of keratohyaline granules. The cyst lining is similar to the surface epithelium but lacks rete ridges which are seen in the overlying epidermis. (B) Foci of rupture are noted and the keratin exposed to the adjacent dermis elicits a neutrophilic and then granulomatous reaction.

On gross pathological examination, the cyst was filled with keratinous material. Histological sections examined demonstrated a benign cystic lesion lined by stratified squamous epithelium with keratinization on the luminal surface (Fig. 2), and there was no evidence of malignancy.

Combining clinical findings, Correlated radiographic imaging, and histopathology, a diagnosis of the presacral epidermoid cyst was finally made.

No postoperative event occurred, and the patient was discharged home on postoperative day 3. On long-term postoperative follow-up, the patient continues to do well, was having regular bowel function, tolerating an age-appropriate diet, and pre-operative pain had improved. Six months postoperatively, there were no signs of recurrence.

## 3 Discussion

Different kinds of cutaneous cysts with fluid or semi-solid content, varying histopathologic characteristics, and clinical importance have been identified. Some cysts have an epithelial cell wall around them that is either stratified or unstratified squamous. These are sometimes referred to as genuine cysts. However, a subclass of cysts known as pseudocysts lacks an epithelial lining and is instead surrounded by connective or granulation tissue. Circular, dome-shaped, projecting, deep-lying, dermal or subcutaneous papules or nodules are frequently observed in different parts of the body as cutaneous cysts. Histopathologic analysis is mostly used to confirm the diagnosis. However, several clinical and imaging characteristics, such as the cyst's location, may offer diagnostic hints that lead to a presumptive diagnosis [9].

A very uncommon congenital lesion of ectodermal origin is the presacral epidermoid cyst. It originates from a piece of ectodermal tissue that develops improperly during embryogenesis as a result of the growth of nearby structures [1]. Presacral epidermoid cysts in men are extremely uncommon, but they have occasionally been observed in asymptomatic women [3]. Although epidermoid cysts can be seen all over the body, they are uncommon in the retrorectal and presacral regions. Although the precise prevalence of presacral epidermoids is unknown, a prior study shows they account for one in 40,000 admissions [10]. The lesions may gradually enlarge with time and develop an infection or inflammation [11]. On histologic examination, these cysts are found to contain a mix of desquamated debris, cholesterol, keratin, and water within a thin wall bordered by stratified squamous epithelium with a characteristic granular cell layer [1].

On the other hand, presacral epidermoid cysts are distinctive in that they are the only embryonic lesions that exhibit diffusion restriction. According to prior reports, incidental observations of presacral epidermoid cysts during gynecologic or obstetric related imaging are typically reported in females of reproductive age group [3]. They tend to stay asymptomatic due to their peculiar position and slow growth. Presence of secondary infection or malignant degeneration has been linked to pain symptoms in presacral lesions [12]. The characteristics of these cysts on computed tomography and ultrasonography are typically difficult to determine. As a result, accurate preoperative diagnosis is challenging. We advise MRI with diffusion weighted imaging for a certain preoperative diagnosis in many lesions to overcome this diagnostic conundrum. Wherever they are located within the body, epidermoid cysts manifest as T1 hypointense, T2 hyperintense masses that exhibit diffusion limitation. Due to the presence of keratin, T2 hypointense foci may be visible within the lesion [3].

An earlier work without discussing diffusion weighted imaging characteristics claimed that presacral epidermoid cyst was preoperatively detected using endorectal ultrasonography, CT, and conventional MRI. They claimed that a diagnosis was made via fine needle aspiration [3]. The risk of seeding malignant cells, if present, and the possibility to secondary infect the cyst's sterile contents make biopsy or needle aspiration generally unnecessary in presacral cystic lesions .

Diffusion weighted imaging on an MRI of the pelvis can be used to define presacral epidermoid cysts, according to a recent case report from Japan [14]. We also found another uncommon characteristic in the diffusion weighted sequences, which involved differential restriction in its composition. On DWI, the posterior portion of the contents had considerably more hyperintense signals, which presumably correlated with keratin deposition. A presacral epidermoid with this appearance has previously been described [14]. Additionally, an MRI can distinguish between bone, spinal canal, meningeal, or symptoms of malignant degeneration [1]. Concerning signs of a malignant alteration include heterogeneous signal intensity on T2, irregularly thickened walls, solid components, and the presence of amplification [13].

A retrorectal pyogenic abscess is the closest differential of an epidermoid cyst based on diffusion limitation. In these situations, a clinical history of prior surgery, a systemic disease, or a high temperature is frequently present. An abscess is typically poorly defined or loculated on imaging, with heterogeneous signals, a thickening enhancing rim, intracavity fluid debris levels, or air particles. Depending on the cause, the surrounding fat may be infiltrated, the rectal wall may be thickened, or there may be signs of diverticulosis. An abscess, which is typically central, may have heterogeneous diffusion restriction on DWI. Additionally, reports have claimed that compared to pelvic cystic tumors, pelvic abscesses typically have lower ADC values [15].

The use of diffusion restriction to describe presacral epidermoid cysts, an uncommon condition, is advantageous for preoperative planning. Due to the potential of subsequent infection or cancer, surgical removal of these lesions is advised. Therefore, it is crucial to get the right diagnosis and start the right course of treatment for a presacral lesion because poor primary surgery might increase morbidity. Additionally, it can raise the possibility of a recurrence and even lead to consequences including fecal incontinence [11].

## 4 Conclusion

Epidermoid cyst is a common entity but is rare at presacral space. Due to the potential of subsequent infection or cancer, surgical removal of these lesions is advised. Diffusion weighted imaging in MRI for characterisation of pelvic lesions should be routinely used now that higher magnetic strength MRI scanners are becoming more common and accessible. Diffusion-weighted MRI can be used to correctly diagnose epidermoid cysts in the presacral/retrorectal region, a rare occurrence. DWI thereby eliminates the necessity for a biopsy, making it a helpful diagnostic technique in this context. It is justified to utilize MRI with diffusion weighted imaging to diagnose pelvic cysts due to the distinctive look of an epidermoid cyst, and this procedure need to be included in the usual pelvic imaging regimen.

## Ethical approval

Informed consent was signed from the patient for publication.

## Authors' contributions

**Study concept or design:** Dr. Fahmi Jubran.

**Writing the manuscript:** Salem M. Tos, Afnan W.M. Jobran, Anas Alasafrah and Izzeddin Bakri.

**Review & editing the manuscript:** Salem M. Tos, Afnan W.M. Jobran.

## Guarantor

Dr. Fahmi Jubran

## Registration of research studies

Not applicable.

## Declaration of competing interest

The authors declare no conflicts of interest.
